# Principles of Surgery

**Published:** 1842-07

**Authors:** 


### Art. XXI.

*Principles of Surgery.* By James Syme, f.r.s.e., Professor of Clinical Surgery in the University of Edinburgh, and Surgeon to the Queen. Third Edition.—*London*, 1842. 8vo, pp. 505, with Plates.

Under the above title, Mr. Syme has published a work which has extended to three editions, a pretty conclusive proof that it is highly estimated. A work may have a certain value because it may be the only one on the subject, and thus, though intrinsically of no great excellence, it may occupy a prominent place; but this is not the case with the work before us: it has several competitors, some more recently written, and with one exception its rivals are all by Mr. Syme's countrymen. We may be accused by our northern brethren of partiality, but we must say that although each may have its merit, we prefer to any of them the humble "First Lines of Surgery" of Mr. Samuel Cooper. When Mr. Syme states that his work is intended to give a comprehensive and systematic view of the facts and opinions which constitute the science of modern surgery, all we can say is that we are astonished at the paucity of those facts and opinions. And when he states that the present edition has been carefully revised and corrected in every point where the improvements of others or his own reflection and experience suggested alteration, we merely say that he has passed a very harsh judgment upon many meritorious discoveries, if he be aware of them, and that if he be not he ought to be.

If we look to the subject of Inflammation, nothing is stated of the observations of Hastings, of Gendrin, of Kaltenbrunner, Macartney, and many others. If we turn to the subject of Suppuration, we have nothing of the curious researches of Henle, Gendrin, Guterböck, Mandl, Müller, Gulliver, and others on pus. In speaking of diseases of blood-vessels, no mention is made of dissecting and some other varieties of aneurism. And we should have thought it would have been proper to say something more of the ligature of the subclavian at its inner third, than this: "the subclavian artery may be tied also on the inner or sternal side of the scalenus, but the numerous branches that spring from the artery here, together with the close neighbourhood of the pleura, vein, and on the left side the thoracic duct, render this operation extremely difficult and dangerous." Would it not have been better to say how many times it had been done, and that it had never succeeded? There are many operations both difficult and dangerous which are performed every week—but is this one that should ever be performed?

These are the kind of drawbacks which are presented in almost every page of his work; and, considering that it is a class-book in the northern capital, and that it is most desirable that the quality of information communicated to the student should be improved, and the standard of education should be raised, we could have wished that more pains had been taken to indicate the sources to which the student should resort for important matter connected with many questions, at present involved in more or less of obscurity.

Mr. Syme is unquestionably an able surgeon, and understands surgery well; but the work before us leaves much to be desired as a text-book for the student. As the work has reached a third edition, it may probably reach a fourth; in that case we would suggest that, if the author's own time is too fully occupied with more important avocations, he should call to his assistance some well-informed young surgeon, who under the author's eye could render very important services in the preparation of the succeeding edition.

